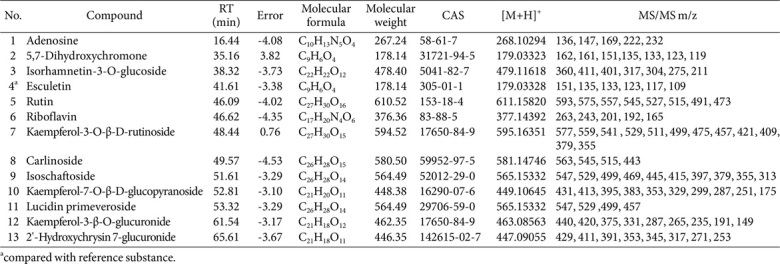# *Viola tianshanica* Maxim Extract Ameliorates Lipopolysaccharide Induced Acute Lung Injury by Regulating NLRP3 Inflammasome and Nrf2 Signaling Pathway

**DOI:** 10.4014/jmb.2501.01052

**Published:** 2025-07-14

**Authors:** Yan Liu, Yuzhu Shi, Lei Xu, Xue Wang

**Affiliations:** Xinjiang Key Laboratory for Uighur Medicine, Xinjiang Institute of Materia Medica, Urumqi 830004, P.R. China

**Keywords:** *Viola tianshanica* maxim extract, acute lung injury, lipopolysaccharide, NLRP3 inflammasome, Nrf2 signaling pathway

## Abstract

Acute lung injury (ALI) is a prevalent critical respiratory disease associated with high morbidity and mortality rates. *Viola tianshanica* Maxim has been traditionally employed in Uygur medicine for treatment of various respiratory diseases. *Viola tianshanica* Maxim extract (VTM) has strong anti-inflammatory and anti-oxidant properties, and its potential protective mechanism against ALI is worthy of further study. In this study, we investigated the protective effect of VTM on lipopolysaccharide (LPS)-induced ALI in mice and explored its underlying mechanisms involving NOD-like receptor protein 3 (NLRP3) inflammasome and Nuclear factor erythroid 2-related factor 2 (Nrf2) signaling pathways. VTM was extracted from *Viola tianshanica* Maxim. Chemical compositions of VTM were identified by HPLC-HRMS/MS. The protective effect and molecular mechanisms of VTM on alleviating ALI were verified by hematoxylin-eosin (H&E) staining, enzyme-linked immunosorbent assay (ELISA), quantitative real-time polymerase chain reaction (qRT-PCR) and Western blot. A total of 13 chemical compositions were analyzed and identified from VTM, including esculetin, isoschaftoside, kaempferol-7-O-β-D-glucopyranoside, among others. VTM effectively alleviated ALI by reducing of the wet-to-dry weight (W/D) ratio, serum inflammatory cytokines PGE2 and LTB4 levels, and lung tissues IL-1β, IFN-γ, TNF-α and MCP-1 levels. VTM significantly decreased mRNA expressions of IL-6, TNF-α and MCP-1, inhibited MDA and MPO formation, and reversed SOD and GSH depletion. Meanwhile, VTM markedly improved histopathological changes, inhibited NLRP3 inflammasome activation by reducing the protein and mRNA expression levels of NLRP3, Caspase 1, GSDMD and IL-1β in lung tissues. Additionally, VTM mitigated oxidative stress in ALI by upregulating the protein and mRNA expression levels of Keap1, Nrf2 and HO-1 in lung tissues. Our findings indicated that pretreatment with VTM prevented LPS-induced ALI by regulating NLRP3 inflammasome and Nrf2 signaling pathway.

## Introduction

Acute lung injury (ALI) is a prevalent and severe acute respiratory disease in clinical practice, characterized by acute onset, rapid progression, critical condition and high mortality rate, posing a significant threat to human life and health [1-3]. A growing body of research indicates that inflammation and oxidative stress are closely related and play a crucial role in the pathogenesis of ALI [4, 5]. Therefore, focusing on the suppression of inflammation and oxidative stress may be a key therapeutic strategy for ALI.

Lipopolysaccharide (LPS) is a main component of the outer cell wall of Gram-negative bacteria. Under LPS stimulation, inflammatory cells rapidly infiltrate the lung tissue, leading to inflammatory cell aggregation and subsequent oxidative stress induction. As a pivotal mechanism of ALI, oxidative stress significantly promotes disease progression and is associated with high mortality rate [6, 7]. The pathogenesis of ALI remains complex and has not been fully elucidated. Inflammation is the most important mechanism in ALI, and excessive inflammatory response plays a crucial role in lung tissue lesions during ALI [8, 9]. NOD-like receptor family pyrin domain containing 3 (NLRP3) inflammasome is a typical inflammatory pathway, which is the core of ALI. The pyroptosis of downstream alveolar macrophages is one of the main causes of lung injury and lung edema [10, 11]. Numerous evidence have demonstrated that oxidative stress critically modulates the pathogenesis and progression of ALI [12, 13]. Nuclear factor erythroid 2-related factor 2 (Nrf2) signaling pathway, a key pathway in cellular oxidative stress response, along with its downstream antioxidant proteins and phase II detoxification enzymes, plays an important role in cell defense and protection. Recent studies have shown the importance of Nrf2 signaling pathway in the occurrence and development of refractory respiratory diseases such as ALI, pulmonary fibrosis, and chronic obstructive pulmonary disease [14, 15].

In recent years, there has been growing attention to the potential of natural products in the prevention and treatment of ALI, which can exert beneficial effects by inhibiting inflammatory response, improving barrier function and alleviating oxidative stress, demonstrating broad application prospects [16, 17]. *Viola tianshanica* Maxim (VTM), the dried whole plant of *Violaceae* species, which is documented in the Pharmaceutical Standards of the Ministry of Health of the People's Republic of China (Uyghur Medicines volume) [18]. It is grown mainly in hillside grasslands, alpine and subalpine meadows, and riparian waterways in Xinjiang Province, China. VTM is widely used for treating conditions such as cold, fever, pleurisy, pneumonia, and pharyngitis. VTM has been extensively applied in traditional Uyghur medicine and is a main component in many classical prescriptions, such as compound *V. tianschanica* Granules, Jiangre Binafuxi Syrup, and Binafuxi Granules. Because of its low side effects and cost, VTM has significant value for further research and development. VTM contains coumarins, flavonoids, volatile oils, and alkaloids, among other components [19, 20]. Studies showed that VTM possesses various pharmacological activities, including anti-inflammatory, antioxidant, antiviral activities and immune regulatory properties [21-24]. However, in-depth research on its primary active ingredients and mechanisms of action is still relatively limited. Previous studies indicate that the maximum dosage of VTM was 4,000 mg/kg in acute toxicity tests, and no animal death and toxic reactions were observed, suggesting a potential for safe use. Furthermore, we found that the ethanol extract of *V. tianshanica* Maxim has protective effect against ALI in mice [25]. Nevertheless, the chemical compositions and the underlying molecular mechanism have not been elucidated. Therefore, the present study aims to further explore the protective effect of VTM on LPS-induced ALI in mice and investigate whether its protective mechanism involves the modulation of NLRP3 inflammasome and Nrf2 signaling pathway.

## Materials and Methods

### Materials and Reagents

Dexamethasone (DEX, SD9530) and Radio immunoprecipitation assay (RIPA) lysis buffer (R0010) were purchased from Solarbio Science & Technology (China). Lipopolysaccharides (LPS, L2880) was provided by Sigma-Aldrich (USA). Mouse IL-1β (88-7013A-88), interferon-gamma (IFN-γ, 88-7314-88), tumor necrosis factor-α (TNF-α, 88-7324-88) and monocyte chemoattractant protein 1 (MCP-1, 88-7391-88) enzyme-linked immunosorbant assays (ELISA) kits were obtained from Thermo Fisher Scientific (USA). Mouse Prostaglandin E2 (PGE2, E-EL-0034) and Leukotriene B4 (LTB4, E-EL-0061) ELISA kits. Myeloperoxidase (MPO, A044-1-1), glutathione (GSH, A006-2-1), superoxide dismutase (SOD, A001-3-2) and malondialdehyde (MDA, A003-1-2) assay kits were supplied by Nanjing Jiancheng Bioengineering Institute (China). TransZol Up kit was offered by TransGen Biotech Co., Ltd., (China). TianGen Biotech Co., Ltd., supplied FastKing gDNA Dispelling RT SuperMix (KR118) and SYBR Green SuperReal PreMix Plus (FP205). Antibodies against Caspase-1 (83383), Nrf2 (12721), ASC (67824) and GAPDH (2118) were products of Cell Signaling Technology (USA). Antibodies against IL-1β (ab254360), Keap1 (ab227828), HO-1 (ab68477) and NLRP3 (ab283819) were obtained from Abcam (UK). Antibody against GSDMD (AF4012) was acquired from Affinity Biosciences (USA). HRP conjugated Goat anti-rabbit IgG (H+L) (ZB-2301) was purchased from Beijing Zhongshan Jinqiao Biotechnology Co., Ltd., (China).

### Preparation of the Extract from *V. tianshanica* Maxim

The plant of *V. tianshanica* Maxim was purchased from Anhui DeChang Pharmaceutical Decoction Pieces Co., Ltd. (China), and identified by Jiang He, a researcher at Xinjiang Institute of Materia Medica. The dried *V. tianshanica* Maxim (5 kg), extracted with 70% ethanol, boiled with water for 3 times, 1.5 h each time, and combined the three filtrate. The 70% ethanol extract was then concentrated under reduced pressure. Subsequently, The extract was extracted respectively with petroleum ether, dichloromethane, ethyl acetate and n-butanol to obtain different polar parts. The n-butanol extraction fraction was purified by AB-8 resin column and eluted with 30%ethanol as the mobile phase, the eluate was collected and concentrated under reduced pressure to obtain the extract of *V. tianshanica* Maxim (VTM, 75 g), with an extraction yield of VTM of 1.5%.

### HPLC-HRMS/MS Analysis

The sample preparation procedure was as follows. Weigh 50 mg VTM and dissolve it in 10 ml 50% ethanol, the solution was sonicated until it completely dissolved and subsequently filtered through a 0.22 μm microporous filter membrane.

The composition analysis of VTM was conducted by HPLC-HRMS/MS, an Ultimate 3000 HPLC and LTQ Orbitrap XL HRMS (Thermo Fisher Scientific). AQ-C18 column (4.6 mm × 250 mm, 3 μm) was used for chromatographic separation. The linear gradient elution of water containing 0.1% (v/v) formic acid (A) and acetonitrile (B). The gradient elution program was as follows: 0-5 min, 0% B; 5-40 min, 0-15% B; 40-85 min, 15-50% B; 85-90 min, 50-0% B. The flow rate was 1.0 ml/min, and the injection volume was 10 μl. The UV detection wavelength was set at 254 nm, and the column temperature was maintained at 25°C.

Mass spectrum analysis adopted the following conditions. Electrospray ionization source (ESI) was used for ionization in positive ion mode, high resolution data acquisition, resolution 30000; ion transfer tube temperature, 370°C; spray voltage, 4.5 kV; ion transfer tube voltage, 60 V; sleeve lens voltage, 80 V; sheath gas, 30 arb; auxiliary gas, 10 arb; purge gas, 0 arb; full Scan mode, scan range (m/z), 50 to 1000. Secondary ions were scanned using data dependence, collision-induced dissociation (CID) data were collected with normalized energy set at 35%.

The mass spectrum data were analyzed using Compound Discoverer 3.3 software, and the application of mass spectrometry data related to the natural product libraries possible material retrieved. The molecular weight deviation of the mass spectrum was set at less than 5 ppm, and the maximum peak area was higher than 1.0 × 10^5^. The obtained retrieved data were filtered to obtain the analysis results in the positive ion mode of the ESI source.

### Animal and Experimental Protocol

Male BALB/c mice (Seven to eight weeks old, 18 to 22 g) were purchased from the Experimental Animal Centre of Xinjiang Medical University (China), production license No. SCXK (Xin) 2018-0002. The mice were allowed free access to food and water in an animal facility with a temperature ranging from 22 to 24°C, relative humidity 40-60% and a 12 h light/dark cycle under specific pathogen-free conditions for a week to adapt to the environment. All animal experiments were approved by the Care & Welfare Committee on Animal Experiments of Xinjiang Institute of Materia Medica (Approval No: XJIMM-20211207) and complied with animal welfare regulations. The methods employed in this study were conducted in accordance with the approved guidelines. The mice were randomly divided into six groups: control group, LPS group (10 mg/kg), LPS + VTM (100 mg/kg) group, LPS + VTM (200 mg/kg) group, LPS + VTM (400 mg/kg) group, and LPS + DEX (positive control, 2.5 mg/kg) group. Doses of VTM were selected based on the previous studies on potent anti-influenza virus pneumonia effects [22]. All drugs were dissolved in 0.5% CMC-Na solution and administered orally for five consecutive days before modeling. One hour after the last administration, mice were anesthetized with diethyl ether, then mice intranasally administered LPS solution (10 mg/kg, dissolved in PBS) to establish the ALI mouse model, except for the control group. Sixteen h after the LPS challenge, mice were anesthetized with sodium pentobarbital (intraperitoneally, 40 mg/kg), blood and lung tissues were collected for subsequent experiments.

### Determination on Lung Wet/Dry Weight (W/D) Ratio

The surface water and blood on lung tissues were absorbed using filter paper. The lung wet weight (W) was measured using an electronic balance. Subsequently, the lung tissues were dried in an oven at 80°C for 48 h, and the dry weight (D) was determined. The W/D ratio was recorded for all groups to evaluate lung swelling.

### Histopathology Evaluation

The lung tissues were soaked in 10% formalin for 24 h. The tissues were dehydrated, embedded in a paraffin block and sectioned to 5 μm. The tissue sections were stained using H&E staining method. Pulmonary injury was measured blindly by two pathologists. Meanwhile, a semi-quantitative scoring method was employed for statistical analysis to compare the differences in histopathological changes of each group. All lung tissue sections were randomly assigned, and the scoring system included three indicators: alveolar hemorrhage, inflammatory cell infiltration, and thickness of alveoli wall. Blind scoring was performed according to the criteria: 0 as none or very minor, 1 as little or limited, 2 as intermediate, 3 as widely distributed or remarkable. The results were scored from 0 to 3 for each item, and three variables were averaged to grade the lung injury score.

### Measurement of Inflammatory Cytokines Levels in Serum and Lung Tissues

The serum was centrifuged at 3,000 rpm for 10 min. The lung tissues were rinsed with pre-chilled PBS to remove blood and dried with filter paper. Then, 1 g of lung was weighed and homogenized by adding 1 ml of PBS. The prepared tissue homogenate was centrifuged at 4°C and 2,500 rpm for 10 min, and the supernatant was retained. Serum PGE2 and LTB4 levels, lung tissues IL-1β, IFN-γ, TNF-α and MCP-1 levels were determined by ELISA kits, according to the recommended instructions. Absorbance values were measured at 450 nm using a Molecular Devices Spectra Max M2 plate reader.

### Measurement of Oxidative-Stress in Lung Tissues

To evaluate the extent of lipid peroxidation resulting from increased oxidative stress, the levels of SOD, MPO, MDA and GSH in lung tissues were detected according to the manufacturer’s instructions of the corresponding kits.

### qRT-PCR Analysis

The total RNA was extracted from the lung tissues using the TransZol Up kit according to the manufacturer’s instructions. The cDNA was synthesized by using FastKing First-Strand RT SuperMix Kit. qRT-PCR was performed by the SYBR Green Premix qPCR kit and specific primers for target genes with the ABI QuantStudio 3 Sequence Detection System. The qRT-PCR conditions were as follows: predenaturation at 95°C for 15 min, denaturation at 95°C for 10 sec, followed by annealing and extension at 60°C for 32 sec. The reference gene GAPDH was used internally in this experiment. Cycle threshold (Ct) value was obtained at the point inflection of the amplification curve, and the relative mRNA expression of the target gene was calculated by 2^-ΔΔCt^ method. The primer sequences are listed in Table 1.

### Western Blot Analysis

Lung tissue samples were lysed sufficiently in RIPA buffer with protease and phosphatase inhibitors for 30 min. The tissue homogenates were centrifuged at 12,000 g for 5 min at 4°C, and the supernatant was collected. Protein concentrations were quantified with a BCA protein assay kit. Total protein was denatured by heating at 95°C for 5 min, and 20 μg of protein was separated by 10% SDS-PAGE and transferred to the PVDF membrane. The membrane was blocked with 5% (w/v) skim milk for 1 h and washed three times with TBST, followed by an overnight incubation at 4°C with a appropriate primary antibodies: NLRP3 (1:1000), Caspase-1(1:1000), GSDMD (1:1000), ASC (1:1000), IL-1β (1:1000), Keap1 (1:2000), Nrf2 (1:1000), HO-1 (1:1000), and GAPDH (1:1000). The following day, membranes were washed three times with TBST, and then incubated with HRP-conjugated IgG secondary antibody (1:10000) for 1 h. After washing three times with TBST, the membranes were exposed an enhanced chemiluminescence (ECL) kit. Finally, band intensities were quantified using Image J gel software.

### Statistical Analysis

All data were analyzed using IBM Statistics SPSS 22.0 and presented as the means ± standard error of the mean (SEM). Comparisons between two groups were performed using a two-tailed Student’s *t*-test, and One-way analysis of variance (ANOVA) was used for comparison between multiple groups. *p* < 0.05 was considered statistically significant. All the graphs were performed by GraphPad Prism 8.0 (GraphPad Software, USA).

## Results

### Composition Analysis of VTM

**Fig. 1:**
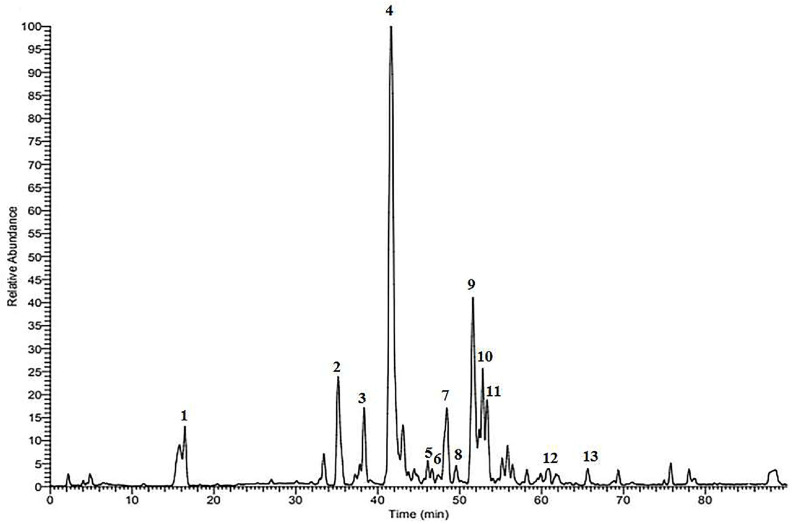
The total ion chromatograms (TIC) of VTM in positive ion mode.

Based on the HPLC-HRMS/MS technique, chemical compositions of VTM were analyzed according to the retention time (RT), molecular fragmentation peaks, molecular weights, molecular formulas, secondary fragmentation and other information, combined with reference substances and literature. As shown in Fig. 1 and Table 2, 13 chemical components of VTM were identified, including 8 flavonoid glycosides, 1 coumarin component, 1 anthraquinone component, and 4 other components. Among them, esculetin was confirmed as the main chemical composition of VTM verified using reference materials.

### VTM Ameliorates LPS-Induced ALI

**Fig. 2:**
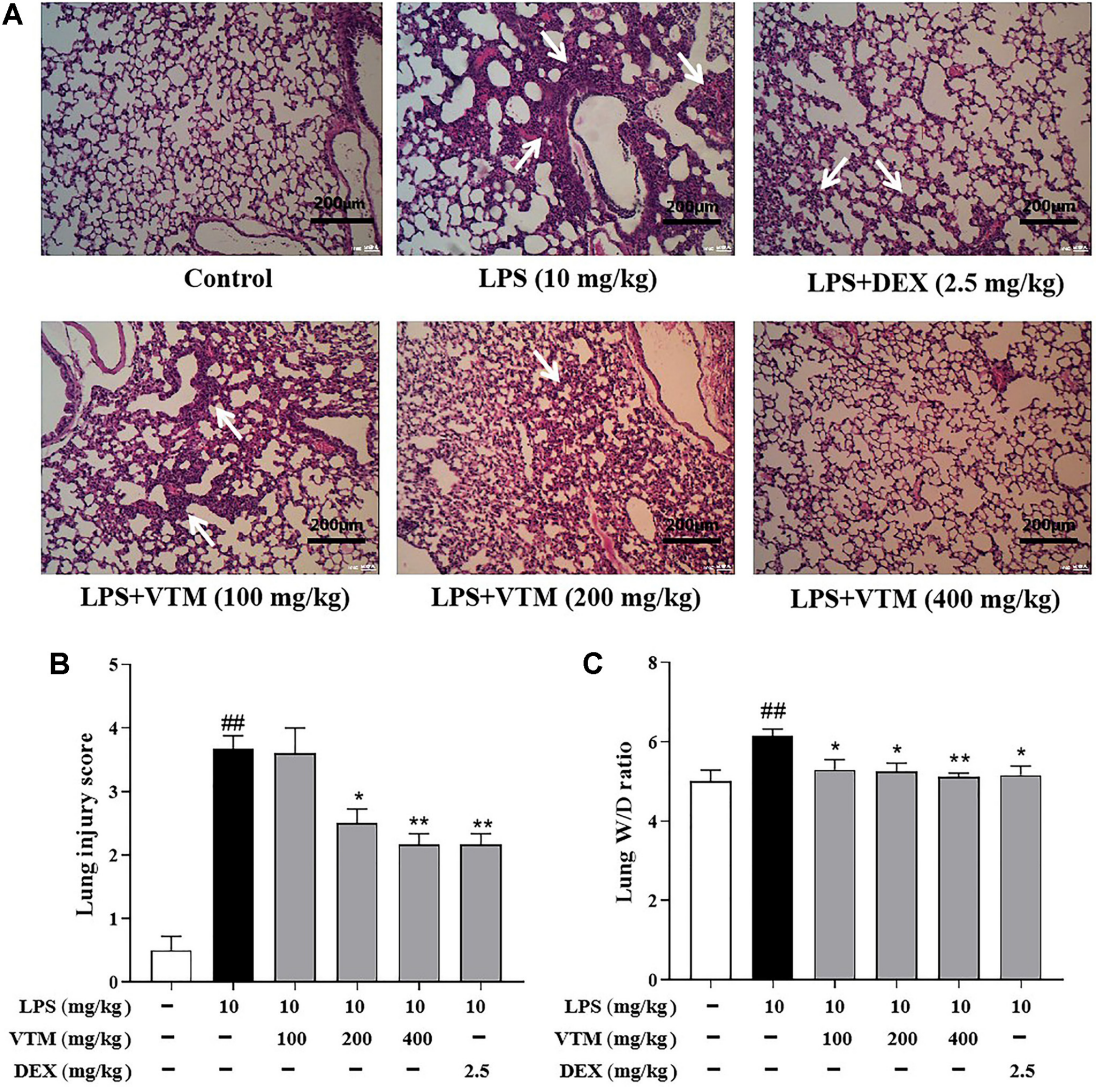
VTM ameliorates LPS-induced ALI. (**A**) The representative lung tissues histological images of mice stained by H&E, Scale bar: 200 μm. (**B**) lung injury score (*n* = 6). (**C**) lung W/D ratio (*n* = 6). Data are expressed as the mean ± SEM. ^#^ *p* < 0.05, ^##^ *p* < 0.01 vs. the control group; *p < 0.05 , **p < 0.01 vs. the LPS group.

Histopathological changes of the lung tissues were assessed using H&E staining. In the control group, lung tissues represented normal structure. Histopathological alterations were found in the LPS group, including alveolar wall thickening, alveolar hemorrhage, alveolar cavity collapse, pulmonary edema, and a large number of inflammatory cells infiltration in the alveolar cavity and lung interstitium (Fig. 2A). Compared with the control group, the LPS group exhibited a higher lung injury score. However, pretreatment with VTM (200, 400 mg/kg) and DEX (2.5 mg/kg) significantly ameliorated LPS induced histopathological changes (Fig. 2B). In order to further clarify the protective role of VTM in LPS-challenged mice, the lung W/D ratio was compared among all groups. Compared with the control group, the lung W/D ratio markedly increased in the LPS group. Compared with the LPS group, pretreatment with VTM (100, 200, 400 mg/kg) significantly decreased the lung W/D ratio (Fig. 2C). These results confirmed the protective effect of VTM against LPS-induced ALI.

### VTM Reduces the Production of Inflammatory Cytokines in ALI Mice

**Fig. 3:**
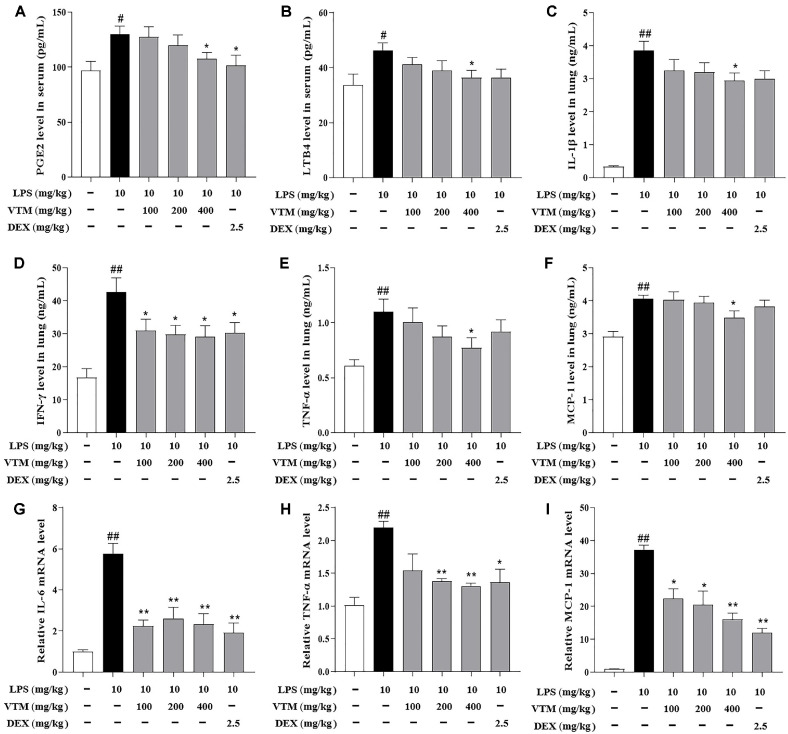
VTM reduces the production of inflammatory cytokines. The levels of inflammatory cytokines PGE2 (**A**) LTB4 (**B**) in serum and IL-1β (**C**), IFN-γ (**D**), TNF-α (**E**) and MCP-1 (**F**) in lung tissues. The mRNA expressions of IL-6 (**G**), TNF-α (**H**) and MCP-1 (**I**) in lung tissues measured by RT-qPCR. The values are presented as the mean ± SEM (*n* = 6). ^#^ *p* < 0.05, ^##^ *p* < 0.01 vs. the control group; *p < 0.05 , **p < 0.01 vs. the LPS group.

To evaluate the effect of VTM on inflammatory responses, the levels of inflammatory cytokines were measured by ELISA. Compared with the control group, mice subjected to LPS significantly increased the production of PGE2, LTB4 in serum (Fig. 3A and 3B), and IL-1β, IFN-β, TNF-α and MCP-1 in lung tissues (Fig. 3C-3F). However, pretreatment with VTM (400 mg/kg) effectively reduced their overproduction, indicating that VTM inhibited inflammation in LPS-induced ALI. Moreover, qRT-PCR analysis showed that mice in the LPS group markedly upregulated mRNA levels of IL-6, TNF-α, and MCP-1. After the VTM intervention, these indicators were significantly reduced (Fig. 3G-3J).

### VTM Alleviates Oxidative Stress in ALI Mice

**Fig. 4:**
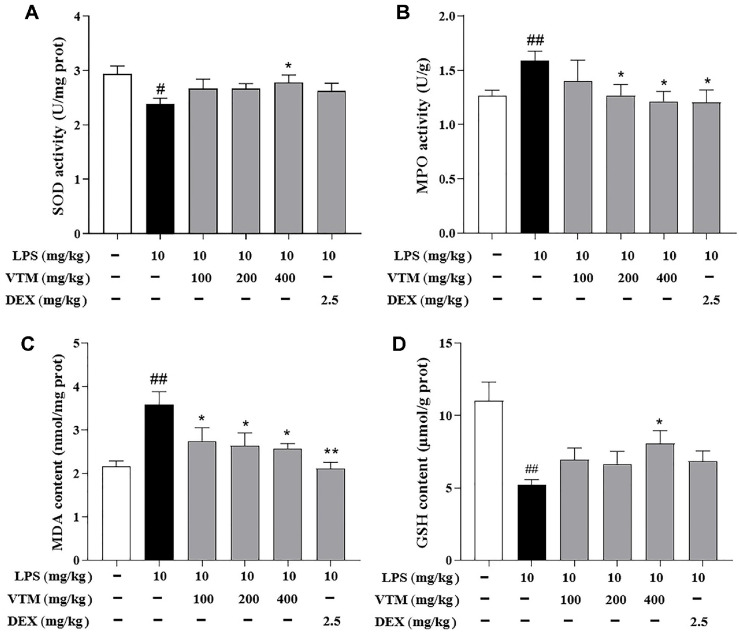
VTM alleviates oxidative stress in ALI mice. (**A**) SOD activity. (**B**) MPO activity. (**C**) MDA content. (**D**) GSH content. Data are expressed as the mean ± SEM (*n* = 6). ^#^ *p* < 0.05, ^##^ *p* < 0.01 vs. the control group; *p < 0.05 , **p < 0.01 vs. the LPS group.

In addition to inflammation, oxidative stress plays a major pathogenic role in LPS-induced ALI. Therefore, we further investigated whether VTM exhibits potential improvement effects against LPS-induced ALI by relieving oxidative stress. Our results showed that LPS-challenge not only significant increased MDA and MPO levels, but also markedly decreased GSH and SOD levels. In contrast, pretreatment with VTM (400 mg/kg) significantly reversed above effects (Fig. 4).

### VTM Inhibits NLRP3 Inflammasome Signaling Pathway in ALI Mice

**Fig. 5:**
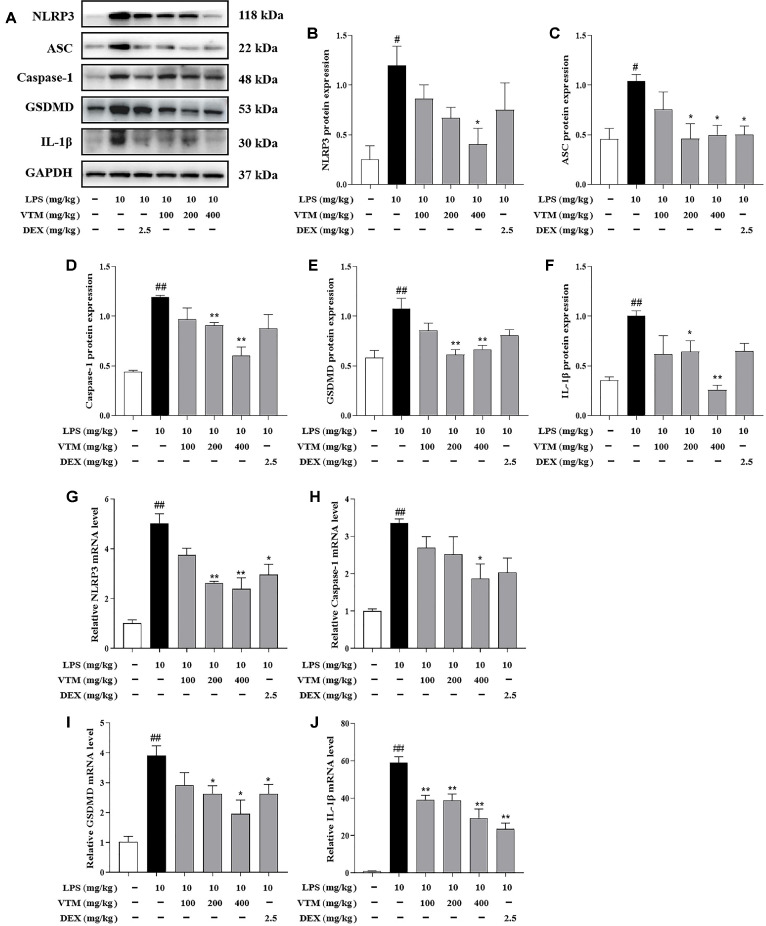
VTM inhibits NLRP3 Inflammasome signaling pathway in ALI mice. (**A**) The protein expressions of NLRP3 (**B**), ASC (**C**), Caspase 1 (**D**), GSDMD (**E**) and IL-1β (**F**) were measured, and GAPDH was used as the internal control by western blot. Statistical analysis of protein abundance. The mRNA expressions of NLRP3 (**G**), Caspase-1 (**H**), GSDMD (**I**) and IL-1β (**J**) were detected by qRT-PCR. Values are expressed as the mean ± SEM (*n* = 3). ^#^ *p* < 0.05, ^##^ *p* < 0.01 vs. the control group; *p < 0.05 , **p < 0.01 vs. the LPS group.

NLRP3 inflammasome is involved in inflammatory processes in a variety of diseases. Currently, inhibition of NLRP3 inflammasome activation is a therapeutic target for treating ALI. Western blot results showed that LPS stimulation remarkably promoted the protein expressions of NLRP3, ASC, Caspase 1, GSDMD and IL-1β, whereas these effects were markedly blunted by pretreatment with VTM (Fig. 5A-5F). Consistently, the mRNA expressions of NLRP3, Caspase-1, GSDMD and IL-1β were analyzed by qRT-PCR, LPS exposure to mice dramatically increased the mRNA expressions of NLRP3, Caspase-1, GSDMD and IL-1β, whereas pretreatment with VTM significantly ameliorated this phenomenon (Fig. 5G-5J). These results indicated that VTM suppressed the activation of NLRP3 inflammasome signaling pathway.

### VTM Regulats Nrf2 Signaling Pathway in ALI Mice

**Fig. 6:**
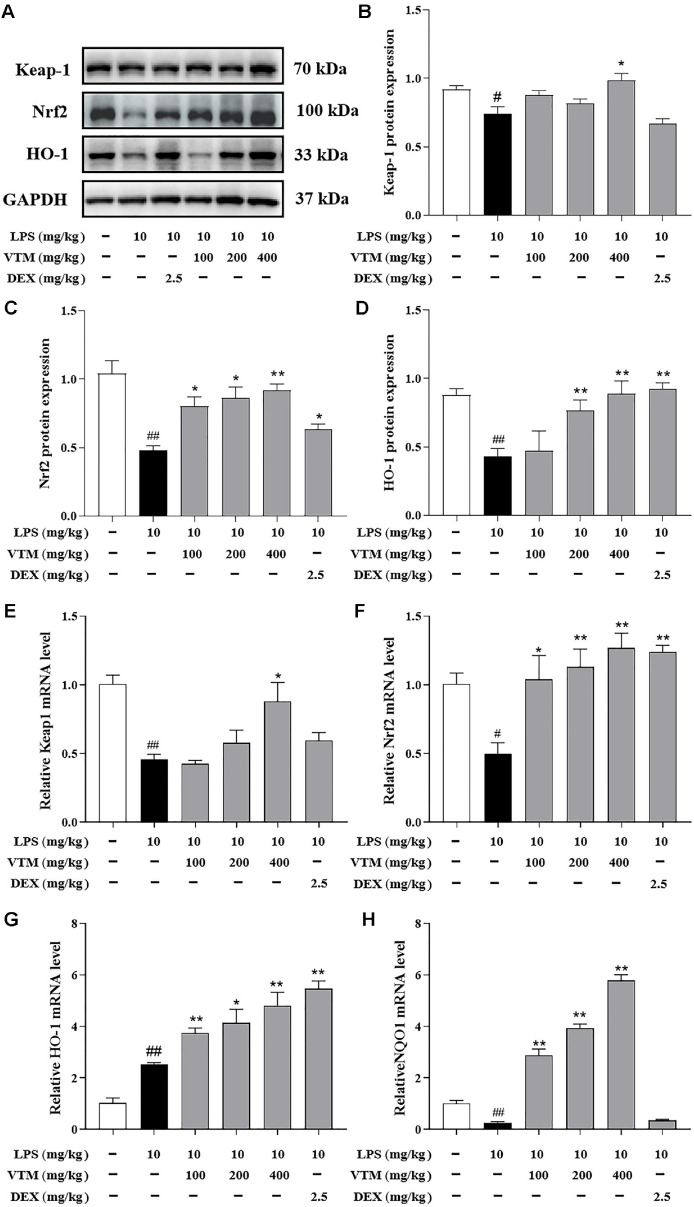
VTM regulats Nrf2/HO-1 and improves the oxidative stress in lung tissues. (**A**) Western blot analysis the protein expression of Keap1 (**B**), Nrf2 (**C**) and HO-1 (**D**) in lung tissues, GAPDH was used as the internal control. Statistical analysis of protein abundance. The mRNA expression of Keap1 (**E**), Nrf2 (**F**) , HO-1 (**G**) and NQO1 (**H**) was measured by qRTPCR. Values are expressed as the mean ± SEM (*n* = 3). ^#^ *p* < 0.05, ^##^ *p* < 0.01 vs. the control group; *p < 0.05 , **p < 0.01 vs. the LPS group.

Nrf2 signaling pathway is involved in the regulation of oxidative stress. To determine whether VTM exerts the protective effect on LPS-induced ALI through Nrf2 signaling pathway, Western blot analysis and qRT-PCR were used to evaluate the protein and gene expression in the lung tissues. Western blot results showed that LPS-challenge decreased the protein expressions of Keap1, Nrf2, and HO-1, but above indexes were all reversed by pretreatment with VTM (Fig. 6A-6D). Additionally, qRT-PCR showed that the mRNA expressions of Keap1, Nrf2, and NQO1 were downregulated in response to LPS exposure, the mRNA expression of HO-1 was increased. However, pretreatment with VTM significantly increased the mRNA expressions of Keap1, Nrf2, HO-1 and NQO1 (Fig. 6E-6H).

## Discussion

ALI is a serious threaten to public health with high morbidity and mortality. Acute respiratory distress syndrome (ARDS), the severe stage of ALI, is a common clinical critical illness. Although enormous advances on the treatment of ALI in recent years, yet no effective medication has been develope. Pathophysiological studies suggest that ALI is primarily caused by inflammation and oxidative stress [26]. Inflammatory cascade reaction and oxidative stress are important driving factors in the occurrence and development of ALI [27]. In patients with severe ALI, uncontrolled inflammation and oxidative stress in the later stage of injury may lead to multiple organ failure. Therefore, anti-inflammatory and antioxidant can be the key strategies for the treatment of ALI.

Natural bioactive ingredients in Chinese herbal medicine have a variety of biological activities, including anti-inflammatory and antioxidant properties, with low toxicity, which are expected to prevent and treat ALI. Our recent study showed that pretreatment with the ethanol extract of *V. tianshanica* Maxim effectively alleviated LPS-induced lung injury by decreasing inflammatory cytokine production and inhibiting neutrophil infiltration in the lung tissues [23]. Nevertheless, whether VTM can improve LPS-induced ALI by regulating NLRP3 inflammasome and Nrf2 signaling pathway is unclear. Therefore, the aim of the present study was to explore the protective mechanism of VTM against inflammation and oxidative stress in LPS-induced ALI mice.

The chemical compositions of VTM were confirmed by HPLC-HRMS/MS analysis, revealing coumarins and flavonoids glycosides compounds as its main constituents. Modern pharmacological experiments demonstrated that most of these compositions exhibit anti-inflammatory and antioxidant activities [28-30]. The underlying mechanisms may involve inhibiting the production of inflammatory mediators, scavenging of free radicals, regulating immune cell functions and cell signaling pathways. Notably, esculetin was identified as the most abundant component in VTM. Several studies demonstrated that esculetin has potent anti-inflammatory property, which is related to its inhibition of NF-κB and MAPKs signaling pathways both *in vitro* and *in vivo* [31]. It has been reported that esculetin ameliorated LPS-induced ALI in mice via modulation of the AKT/ERK/NF-κB and RORγt/IL-17 pathways [32]. Additionally, esculetin attenuated alveolar injury and fibrosis induced by the interaction between alveolar epithelial cells and blood-derived macrophages via IL-8 signaling [33]. Recent studies also revealed that esculetin could protect C2C12 myoblasts against oxidative stress-induced injury, possibly through the activation of the Nrf2/NQO1 pathway [34].

An appropriate experiment model is essential for elucidating the underlying pathophysiological mechanisms and evaluating the therapeutic interventions of ALI. LPS induced ALI animal model is widely used in ALI research [28]. In our study, we established a mouse ALI model by nasal infusion of LPS to observe the protective effect of VTM. Pulmonary edema is a central pathological feature of ALI, and the lung W/D ratio was used to evaluate the degree of pulmonary edema. Our results showed that the lung W/D ratio in the model group was significantly increased. Pretreatment with VTM remarkably decreased the lung W/D ratio, indicating decreased pulmonary edema. H&E staining can better reflect the structure and cell morphology in lung tissues, so it was employed to observe the pathological changes in lung tissues. H&E staining showed significant thickening of alveolar wall, alveolar hemorrhage, collapse of alveolar cavity, and extensive inflammatory cell infiltration in alveolar cavity and lung interstitium in the LPS group. After VTM intervention, the lung injury score was significantly reduced, and the degree of lung tissue injury was markedly lessened. These results indicate that VTM could improve the pulmonary pathological changes in LPS-induced ALI.

Dexamethasone (DEX) is a widely used glucocorticoid with significant anti-inflammatory effects. Several studies have demonstrated that DEX effectively mitigates pathological changes and inflammatory responses in ALI [35]. For instance, DEX inhibits the expression of pro-inflammatory factors such as TNF-α and IL-6 by binding to glucocorticoid receptors (GR), thereby reducing the infiltration of inflammatory cells into lung tissue. Additionally, DEX alleviates pulmonary edema by decreasing the lung W/D ratio and vascular permeability. Histopathological scoring further confirms that DEX attenuates key injury features, including alveolar septal thickening and inflammatory cell infiltration [36]. In multiple studies, the therapeutic efficacy of DEX has been frequently employed as a gold standard for comparison with other candidate drugs. Our findings suggested that VTM has comparable efficacy to DEX in ameliorating ALI.

The inflammatory reaction plays a critical role in the occurrence and development of ALI [37]. LPS induces a large number of inflammatory cells to infiltrate and release in the lung tissues. Excessive inflammatory cytokines and sharply increased inflammatory cytokines, triggering an inflammatory storm through cascade reaction, causing apoptosis of pulmonary capillary endothelial cells and alveolar epithelial cells, resulting in severe injury or even loss of lung function [38, 39]. Therefore, timely regulation of pulmonary inflammatory response may be an effective strategy to treat ALI and improve prognosis. ELISA results showed that serum PGE2 and LTB4 levels, lung tissues TNF-α, IL-1β, IFN-γ and MCP-1 levels significantly increased in the LPS group. Further qRT-PCR analysis revealed that the mRNA expression levels of IL-1β, IL-6, TNF-α, MCP-1 and NF-KB in lung tissues significantly increased. However, pretreatment with VTM reversed the above indexes, indicating that VTM could inhibit the release of excessive inflammatory cytokines in ALI mice, and thus play a protection role in ALI.

Oxidative stress resulting from an imbalance between oxidation and antioxidation, is a crucial mechanism in the development of ALI. Some oxidants act as inflammatory signaling molecules, activating major inflammatory pathways such as NLRP3 inflammasome, leading to the exacerbation of the inflammatory response. MDA, a product of lipid peroxidation, directly reflects the level of free radicals and the extent of lipid peroxidation in biological membranes [40]. Meanwhile, MPO is primarily generated by neutrophil secretion and leads to oxidative damage. SOD functions as a peroxidase within cells, responsible for scavenging oxygen free radicals [41, 42], whereas GSH plays a role in detoxification and antioxidation processes. Imbalance in oxidation/antioxidation system lead to abnormal activation of signal transduction pathways, cell apoptosis, and excessive lung injury [43]. Therefore, besides anti-inflammatory therapy, it is essential to actively control oxidative stress response and adjust these indicators to effectively mitigate ALI. In this study, pretreatment with VTM significantly reduced the levels of MPO and MDA and significantly increased the levels of SOD and GSH in lung tissues, indicating that anti-oxidative stress is a pivotal mechanism to alleviate LPS-induced ALI.

It is noteworthy that multiple signaling pathways are activated or inhibited, which are closely related to the regulation of inflammatory response and oxidative stress in process of LPS-induced ALI [44, 45]. Pyroptosis is an intracellular inflammatory signaling pathway activated by exogenous stimuli, which is accompanied by the formation of inflammasomes assembled by the innate immune receptor protein NLRP3, the aptamer protein ASC, and the inflammatory protease Caspase-1, while activating Caspase-1, thereby inducing GSDMD-dependent programmed cell death [13]. Studies have confirmed that NLRP3 inflammasome is a key link in the initiation of pyroptosis [46, 47]. Generally, macrophage pyroptosis significantly aggravates the progression of ALI/ARDS primarily through exacerbating pulmonary inflammation and tissue damage [48]. In recent years, a large number of studies have shown that direct or indirect inhibition of NLRP3 inflammasome-mediated macrophage pyroptosis reduces the release of interleukin-1β (IL-1β) and IL-18, inhibit inflammatory response, and alleviate tissue damage in ALI mice [49, 50]. This study found that the protein expressions of NLRP3, ASC, Caspase-1, GSDMD and IL-1β and the mRNA expressions of NLRP3, Caspase-1, GSDMD and IL-1β in the lung tissues of ALI mice significantly increased. However, VTM intervention could obviously reverse the above changes. The above results suggested that VTM could play a protective role in ALI by inhibiting NLRP3/Caspase-1/GSDMD mediated pyroptosis. Nrf2 signaling pathway is an important signaling pathway for maintaining oxidative stress and redox balance in the body [51, 52]. Nrf2 is an vital transcription factor responsible for regulating oxidative stress response [53, 54]. Under normal physiological conditions, Keap1 and Nrf2 combine into a complex, inhibit the presence of the state in the cytoplasm, and bind to Cul3. Therefore, Nrf2 is continuously ubiquitinated and degraded by the proteasomeand, and the Nrf2 content *in vivo* remains at a low level. When the body is under oxidative stress, the conformation of cysteine residues in Keap1 protein structure changes, and then uncouples with Nrf2 [55, 56]. Free Nrf2 enters the nucleus and binds with ARE, regulates the expression of downstream antioxidant proteins and detoxicating enzymes, and transcribes HO-1, NQO1, etc. [57, 58]. Consequently, oxidative stress can reduce the damage to cells and tissues [59, 60]. Nrf2 deficiency leads to severe lung damage in mice exposed to LPS. On the contrary, it has been reported that increased Nrf2 protein levels reduced LPS-induced lung injury in mice, and the protective effect of drugs is associated with the activation of Nrf2 signaling pathway [61, 62]. Consistent with previous reports, we found that VTM pre-administration could significantly inhibit Nrf2 protein degradation and promote downstream HO-1 protein transcription, thus exerting an anti-oxidative stress effect.

Increasing evidence indicates that the activation of the Nrf2 signaling pathway is correlated with the inhibition of NLRP3 inﬂammasome activation at multiple levels [63, 64]. Nrf2 knockdown significantly increases the mRNA level of NLRP3. In addition, upregulating of AMPK phosphorylation can promote expression of Nrf2 via inhibition of NLRP3 transcription, thereby inhibiting pyroptosis in alveolar macrophages and ultimately reducing ALI [65]. Melatonin has been reported to alleviate ALI *in vivo* and *in vitro* by inhibiting NLRP3-GSDMD pathway through activating Nrf2/HO-1 signaling pathway [66]. In conclusion, inhibition NLRP3 inflammasome activation by upregulating Nrf2 level is important for alleviating ALI. The mechanism not only involves antioxidant and anti-inflammatory effects, but also may achieve comprehensive regulation of inflammatory response by regulating Nrf2 signaling pathway. Our results suggest that VTM showed a protective effect in LPS-induced ALI via regulating NLRP3 inflammasome and Nrf2 signaling pathway, which was consistent with previous reports.

In recent years, the rapid progress in molecular biology techniques has greatly facilitated experimental research of Chinese herbal medicine for ALI prevention and treatment. Nevertheless, research on the mechanisms is still limited, mostly focusing on initial exploration such as inﬂammatory cell inﬁltration, and oxidative stress regulation. Deeper mechanistic insights and comprehensive investigations remain to be fully explored. In this study, we have only conducted a preliminary investigation on the efficacy and mechanistic of VTM against ALI. It is clear that VTM alleviates ALI via NLRP3 inflammasome and Nrf2 signaling pathway, but further studies are required to reveal the underlying mechanism, including the use of inhibitors and knockout mice in the future. In addition, the long-term use of VTM along with the adverse effects and the impact on patients requires additional research.

## Conclusion

In conclusion, our results firstly demonstrated that VTM has a protective effect against inflammation damage and oxidative stress in LPS-induced ALI. The beneficial effect of VTM was closely related to the regulation of NLRP3 inflammasome and Nrf2 signaling. Hence, this study provides experimental evidence for the application of VTM in the prevention and treatment of diseases related to inflammation and oxidative stress, especially ALI.

## Figures and Tables

**Table 1:** Primer sequences used in Real-time PCR.

Gene	Forward Primer (5' to 3')	Reverse Primer (5' to 3')
IL-1β	CAACTGCACTACAGGCTCCG	GTGGGTGTGCCGTCTTTCAT
IL-6	AGACAAAGCCAGAGTCCTTCAG	AGGAGAGCATTGGAAATTGGG
TNF-α	ACGGCATGGATCTCAAAGACA	GTGAGGAGCACGTAGTCGG
MCP-1	TTAAAAACCTGGATCGGAACCAA	GCATTAGCTTCAGATTTACGGGT
NLRP3	GCTGCGATCAACAGGCGAGAC	CCATCCACTCTTCTTCAAGGCTGTC
Caspase-1	ATACAACCACTCGTACACGTCTTGC	TCCTCCAGCAGCAACTTCATTTCTC
GSDMD	ACTGAGGTCCACAGCCAAGAGG	GCCACTCGGAATGCCAGGATG
Keap1	TGCTCAACCGCTTGCTGTA	ATCATCCGCCACTCATTCCTC
Nrf2	ATGATGGACTTGGAGTTGCC	ACTTGTACCGCCTCGTCTG
HO-1	CCGCCTTCCTGCTCAACAT	CTGACGAAGTGACGCCATCT
NQO1	ATGAAGGAGGCTGCTGTAGAG	AGATGACTCGGAAGGATACTGAA
GAPDH	GGCAAATTCAACGGCACAGTCAAG	TCGCTCCTGGAAGATGGTGATGG

**Table 2:** HPLC-HRMS/MS data of compounds of VTM in positive ion mode.